# Association of adiposity with risk of obstructive sleep apnea: a population-based study

**DOI:** 10.1186/s12889-023-16695-4

**Published:** 2023-09-21

**Authors:** Hai Deng, Xueru Duan, Jun Huang, Murui Zheng, Miaochan Lao, Fan Weng, Qi-ying Su, Zhen-feng Zheng, Yunting Mei, Li Huang, Wen-han Yang, Xiaohui Xing, Xiaofeng Ma, Wenjing Zhao, Xudong Liu

**Affiliations:** 1 Department of Cardiology, Guangdong Cardiovascular Institute, Guangdong Provincial People’s Hospital, Guangdong Academy of Medical Science, Southern Medical University, Guangzhou, 510080 China; 2 Department of Epidemiology, School of Public Health, Sun Yat-Sen University, Guangzhou, 510080 China; 3 School of Public Health, Guangdong Pharmaceutical University, 283 Jianghai Avenue, Haizhu District, Guangzhou, 510310 China; 4 Department of Geriatrics, Guangdong Provincial Geriatrics Institute, Guangdong Provincial People’s Hospital, Guangdong Academy of Medical Science, Southern Medical University, Guangzhou, 510080 China; 5 Faculty of Health Sciences, University of Macau, Macau SAR, 999078 China; 6 Department of Sleep Center, Department of Geriatric Respiratory, Guangdong Provincial Geriatrics Institute, Guangdong Provincial People’s Hospital, Guangdong Academy of Medical Sciences, Guangzhou, 510080 China; 7 Guangzhou Yuexiu District Center for Disease Control and Prevention, Guangzhou, 510080 China; 8 Dadong Street Community Health Service Center, Guangzhou, 510080 China; 9 Nancun Community Health Service Center, Guangzhou, 511442 China; 10 Guangdong Provincial Engineering Technology Research Center of Public Health Detection and Assessment, School of Public Health, Guangdong Pharmaceutical University, Guangzhou, 510310 China; 11 Qinghai Province Cardiovascular and Cerebrovascular Disease Specialist Hospital, 7 Zhuanchang Road, Xining, 810012 China; 12 School of Public Health and Emergency Management, Southern University of Science and Technology, Nanshan District, 1088 Xueyuan Avenue, Shenzhen, 518055 China

**Keywords:** Adiposity, Abdominal obesity, Obstructive sleep apnea

## Abstract

**Background:**

Obesity is a crucial risk factor for obstructive sleep apnea (OSA), but the association between adiposity deposition and OSA risk has not reached a consistent conclusion. This study sought to reveal the association of multiple adiposity indicators with OSA risk.

**Methods:**

This cross-sectional study included 9,733 participants aged 35–74 years, recruited from an ongoing population-based cohort. OSA was assessed by the Berlin Questionnaire. Six adiposity indicators, including neck circumference (NC), body fat percentage (BF%), waist-to-hip ratio (WHR), visceral adiposity index (VAI), lipid accumulation product (LAP), and resting metabolic rate (RMR), were selected. Multivariate logistic regression models were used to examine the association of adiposity indicators with OSA risk.

**Results:**

One thousand six hundred twenty-six participants (16.71%) were classified into the OSA group. NC, BF%, WHR, VAI, LAP, and RMR were all positively associated with the risk of OSA after adjusting for confounders, regardless of age, sex, and history of dyslipidemia. Every 1-unit increment of NC, BF%, and VAI was associated with a 13%, 9%, and 14% increased risk of OSA, respectively; every 0.01-unit increment of WHR was associated with a 3% increased risk of OSA; every 10-unit increment of LAP and RMR was associated with 2% and 4% increased risk of OSA, respectively.

**Conclusions:**

NC, BF%, WHR, VAI, LAP, and RMR were all independently and positively associated with OSA risk, regardless of age, sex, history of dyslipidemia, and menopausal status. Application of these new indicators could help to more comprehensively reflect and predict the risk of OSA in the general population.

**Supplementary Information:**

The online version contains supplementary material available at 10.1186/s12889-023-16695-4.

## Introduction

Obstructive sleep apnea (OSA) is a common and under-recognized sleep disorder, characterized by periodic reductions or cessations in ventilation caused by dependent complete or partial collapse of the upper airway, resulting in consequent hypoxia, hypercapnia, or arousals from sleep [1]. OSA has affected 9% to 38% of the general adult population in Europe and North America [2], 14.0% to 39.4% in Asia [3], and 8.8% to 24.2% in China [4]. It is estimated that only about 1 in 50 patients with symptoms suggestive of OSA syndrome is evaluated and treated [5], as quite a few OSA patients are under-diagnosed or asymptomatic [6]. When left untreated, individuals with OSA are at heightened risk of metabolic syndrome, cardiovascular diseases, reduced quality of life, premature death, etc. [1, 6].

Obesity is one of the most important risk factors for OSA [1], and weight change can influence OSA severity [7]. Body mass index (BMI) is a traditional indicator of general obesity and is widely used in predicting OSA [8]. However, BMI has been criticized for failing to distinguish the fat distribution [9], because OSA is mainly associated with the central distribution of body fat [10]. Waist-to-hip ratio (WHR), an indicator of abdominal obesity, has been demonstrated more strongly linked with OSA than BMI [11]. Most adult OSA patients have abdominal obesity and increased visceral fat, releasing more inflammatory cytokines than peripheral obesity with predominant subcutaneous fat accumulation [7, 11]. This could lead to neck adiposity, increased upper airway fat, and metabolic abnormalities, even in normal-weight subjects [7]. A cross-sectional study among 1,912 Turkish adults showed that neck circumference (NC) was significantly associated with OSA risk, and its ability to predict OSA was greater than that of waist circumference (WC) [12].

Visceral adiposity index (VAI) and lipid accumulation index (LAP) are newly proposed indicators combining anthropometric indicators with lipid levels. The former is a sensitive indicator to reflect visceral obesity, and the latter is derived from the combination of triglyceride level and waist circumference [13]. Zou and colleagues found that LAP and VAI were moderately correlated with OSA severity, and suggested that anthropometry combined with visceral fat markers could be a more effective diagnostic tool for OSA [13]. Besides, body fat percentage (BF%) is commonly used in obesity research, but there are few studies on its relationship with OSA. A study in Uppsala found that men with severe OSA had a higher BF% than those without OSA, even if the cases and controls were matched for age and BMI [14]. Also, considering that obesity is the result of energy imbalance and the resting metabolic rate (RMR) is correlated with daily energy expenditure, it would be more useful to combine RMR with adiposity indicators to explore the relationship between obesity and OSA.

However, the single utilization of the aforementioned indicators could not adequately reflect the effect of adiposity on OSA risk and current studies have not yielded consistent conclusions. Less is known about the association of novel indicators (such as VAI and LAP) with the risk of OSA. Therefore, this large-scale study was conducted by considering NC, WHR, VAI, LAP, BF%, and RMR to examine the association of adiposity with OSA risk based on Chinese adults.

## Methods

### Setting and subjects

This cross-sectional study was based on the Guangzhou Heart Study, an ongoing population-based prospective cohort. The baseline survey was accomplished from 2015 to 2017 in Guangzhou permanent residents by multistage sampling method. The details have been described elsewhere [15–18]. In brief, a total of 12,013 participants aged ≥ 35 years were recruited in the baseline survey, and 2,280 subjects were excluded due to the following exclusion criteria: age older than 74 years (*n* = 1,043), lack of OSA-related data (*n* = 5), suffering from the chronic obstructive pulmonary disease (COPD, *n* = 678) or cardiovascular disease (CVD, *n* = 554). Recent studies have demonstrated that COPD characterized by a chronic bronchitis phenotype could promote OSA, while lung hyperinflation could protect against OSA [19]. OSA patients tend to be comorbid with CVD [20], which may affect the reliability of our results. Therefore, participants with COPD or CVD were excluded to avoid potential bias. Ultimately, 9,733 participants were selected for further analyses. This study was approved by the Ethical Review Committee for Biomedical Research, School of Public Health, Sun Yat-sen University. The study was performed following the Declaration of Helsinki and written informed consent was obtained from each participant.

### OSA ascertainment

OSA was determined by the Berlin Questionnaire (BQ), which was widely used to screen for OSA [21]. The Chinese versions of BQ have been proven to have superior predictive validity and reliability [22, 23]. BQ is a commonly used questionnaire in epidemiological and clinical settings and consists of ten questions in three categories: snoring and breathing cessation (Category 1), excessive daytime sleepiness (Category 2), and BMI and hypertension (Category 3). Category 1 and Category 2 are considered positive with a persistent report of corresponding symptoms (frequency more than three times per week), and Category 3 is considered positive with the report of a history of hypertension or with a BMI of more than 30 kg/m^2^. Positive scores in two or more categories suggest that the respondent is at high risk for OSA, otherwise at low risk [24]. Then the participants judged to be at high risk of OSA by BQ were assigned to the OSA group and those at low risk of OSA were assigned to the non-OSA group.

### Adiposity indicators and anthropometric measurements

Six adiposity indicators were assessed, including NC, BF%, WHR, RMR, VAI, and LAP. Each participant was asked to wear light clothes and step barefoot on the uniformed device to undergo a physical measurement by trained staff. Height and weight were measured to the nearest 0.1 cm (cm) and 0.1 kg (kg), respectively. NC, WC, and hip circumference (HC) were measured to the nearest 0.1 cm through a portable measuring tape. Subjects were asked to stand upright and look straight ahead with shoulders down, and NC was measured by putting the measuring tape midway around the neck, at the level of the laryngeal prominence. WC was gauged at the midpoint between the iliac crest and the lower end of the rib cage, and HC was measured at the maximum extension of the buttocks. Height, weight, NC, WC, and HC were all measured three consecutive times and the mean of each parameter was calculated. BMI was calculated as the mean of body weight in kilograms divided by the mean of height in meters squared (kg/m^2^) and WHR was calculated by dividing the mean measurement of WC by that of HC.

BF%, VAI, and RMR were measured by the bioelectrical impedance device (OMRON-HBF-371-SH: OMRON Corporation, Yangzhou, China) [25]. BF% was calculated by dividing total fat mass by total mass (including fat mass and fat-free mass) and then multiplying by 100. LAP is based on a combination of waist circumference and the fasting concentration of circulating triglycerides and is defined to describe the extent to which an individual has traveled the route of both increasing waist and increasing triglycerides [26]. LAP is calculated depending on gender: LAP for men = (WC [cm]—65) × (triglycerides concentration [mmol/L]), LAP for women = (WC [cm]—58) × (triglycerides concentration [mmol/L]). To avoid having nonpositive values for LAP, any waist values for men that were 65 cm or less were revised upward to 66.0 cm and for women that were 58 cm or less were revised upward to 59.0 cm [26].

### Potential confounding factors

Structured questionnaires were applied to acquire information on demographic characteristics, lifestyle factors, and history of diseases at the face-to-face interview. The modified Global Physical Activity Questionnaire was used to assess leisure-time physical activity (LTPA, MET-h/week) for each participant as we reported previously [15]. Blood pressure was measured and serum cholesterol, low-density lipoprotein cholesterol, and triglyceride were detected. The participant who self-reported physician-diagnosed dyslipidemia or with serum cholesterol of ≥ 5.2 mmol/L, or low-density lipoprotein cholesterol of ≥ 3.4 mmol/L or triglyceride of ≥ 1.7 mmol/L was defined as having dyslipidemia [27]. The subject who self-reported physician-diagnosed hypertension or whose systolic blood pressure was ≥ 140 mmHg or diastolic blood pressure ≥ 90 mmHg was considered as having hypertension. The confounders included age (years), sex (male, female), marital status (married, others), educational status (primary school and lower, junior high school, senior high school, and college or above), work intensity (light, moderate, vigorous, and retirement), smoking (never, occasion or frequent smoking), alcohol drinking (never, occasion or frequent drinking), vegetable intake (< once/day, ≥ once/day), fruit intake (< once/day, ≥ once/day), hypertension (yes, no), and dyslipidemia (yes, no).

### Statistical analysis

All statistical analyses were performed using R software (version 3.6.3). Data were expressed as mean (standard deviation, SD), median (interquartile range, IQR), or frequency (proportion, %), in accordance with the normal, skewed, or categorical distribution. Differences in the baseline characteristics among the non-OSA and OSA groups were computed by t-test, Wilcoxon rank-sum test, or chi-square test. The Pearson correlation test was used for the normally distributed data, and the Spearman correlation test was used for the non-normally distributed data. Each adiposity indicator was converted to a categorical variable based on the quartiles.

The odds ratio (OR) with a 95% confidence interval (CI) was calculated using logistic regression models to demonstrate the association between each indicator and OSA risk. Three models were considered: model 1 was without any adjustment; model 2 was adjusted for age, sex, marital status, education, smoking, alcohol drinking, fruit intake, vegetable intake, work intensity, and LTPA; model 3 was further mutually adjusted for adiposity indicators, aiming to examine the independent association of each indicator with OSA. The multicollinearity was also considered among all variables in the models and variance inflation factors (VIFs) were calculated. The results showed that BMI was not suitable for the adjusted models (VIF > 10) because BMI was closely correlated with adiposity indicators.

Stratified analysis was conducted by age (< 65 years, ≥ 65 years), sex (male or female), and dyslipidemia (yes, no). The multiplicative interaction of adiposity indicators with age, sex, and dyslipidemia was calculated, with the likelihood ratio test by comparing the likelihood scores of the two models with or without the interaction items. A sensitivity analysis was conducted by adjusting the upper and lower 2.5% of the adiposity indicators to the means of which, aiming to exclude the influence of possible outliers. Besides, considering that women's menopausal status plays an important role in OSA occurrence [28], we divided all women into premenopausal group and postmenopausal group based on their self-reported information. Then, we repeated analyses to estimate whether there were differences in the associations between adiposity indicators and OSA risk in women with different menopausal status. All *P* values were two-tailed and a *P* value < 0.05 was considered statistically significant.

## Results

A total of 9,733 participants were enrolled in this study and 1626 participants (16.71%) were classified into the OSA group. Relative to the participants in the non-OSA group, subjects in the OSA group were more likely to be older, male, and married, to smoke and drink alcohol, to be retirees or take up a vigorous occupation, to have a higher level of education, to eat vegetables or fruit at least once per day, to actively take up LTPA, to have hypertension or dyslipidemia, to have a higher value of BMI, NC, BF%, WHR, VAI, LAP, and RMR (Table 1).

**Table 1:** Basic characteristics of the study participants

Characteristics	Non-OSA (*N* = 8107)	OSA (*N* = 1626)	*P* value
Age, year, mean (S.D.)	55.48 (9.98)	58.53 (8.93)	< 0.001^*^
Sex (%)			< 0.001^†^
Male	2469 (30.46)	804 (49.45)	
Female	5638 (69.54)	822 (50.55)	
Marital status, marital, N (%)			< 0.001^†^
Married	7099 (87.57)	1476 (90.77)	
Others	1008 (12.43)	150 (9.23)	
Educational status, N (%)			0.002^†^
Primary school and lower	2879 (35.51)	590 (36.29)	
Junior high school	2081 (25.67)	397 (24.42)	
Senior high school	2043 (25.20)	462 (28.41)	
College and above	1104 (13.62)	177 (10.89)	
Smoking, N (%)			< 0.001^†^
Never	6605 (81.47)	1160 (71.34)	
Occasion	329 (4.06)	132 (8.12)	
Frequent	1173 (14.47)	334 (20.54)	
Alcohol drinking, N (%)			< 0.001^†^
Never	6404 (78.99)	1168 (71.83)	
Occasion	1268 (15.64)	305 (18.76)	
Frequent	435 (5.37)	153 (9.41)	
Work intensity, N (%)			< 0.001^†^
Light	2801 (34.55)	429 (26.38)	
Moderate	888 (10.95)	163 (10.02)	
Vigorous	460 (5.67)	91 (5.60)	
Retirement	3958 (48.82)	943 (58.00)	
Vegetable intake, N (%)			0.049^†^
< once/day	280 (3.45)	73 (4.49)	
≥ once/day	7827 (96.55)	1553 (95.51)	
Fruit intake, N (%)			< 0.001^†^
< once/day	2837 (34.99)	661 (40.65)	
≥ once/day	5270 (65.01)	965 (59.35)	
Hypertension, yes, N (%)			< 0.001^†^
No	5669 (69.93)	157 (9.66)	
Yes	2438 (30.07)	1469 (90.34)	
Dyslipidemia, yes, N (%)			< 0.001^†^
No	2497 (30.80)	414 (25.46)	
Yes	5610 (69.20)	1212 (74.54)	
LTPA, MET-h/week, median (IQR)	35.70 (17.80, 59.20)	34.70 (15.50, 58.80)	< 0.001^*^
WHR, median (IQR)	0.87 (0.82,0.92)	0.91 (0.87,0.96)	< 0.001^‡^
VAI, median (IQR)	7.00 (5.00, 10.00)	11.00 (8.00, 15.00)	< 0.001^‡^
LAP, median (IQR)	28.90 (16.71, 47.76)	44.70 (27.80, 69.15)	< 0.001^‡^
RMR, Kcal/day, median (IQR)	1260.00 (1151.00,1431.50)	1435.00 (1277.00,1607.75)	< 0.001^‡^
BMI, kg/m^2^, mean (S.D.)	23.54 (3.26)	26.34 (3.84)	< 0.001^*^
NC, cm, mean (S.D.)	34.17 (3.05)	36.59 (3.45)	< 0.001^*^
BF%, mean (S.D.)	30.18 (6.19)	31.40 (6.23)	< 0.001^*^

*Abbreviation*: *LTPA* Leisure-time physical activity, *LAP* Lipid accumulation product, *VAI* Visceral adiposity index, *RMR* Resting metabolic rate, *WHR* Waist-to-hip ratio, *BMI* Body mass index, *NC* Neck circumference, *BF%* Body fat percentage

^*^ *P* value from t test

^†^ *P* value from chi-square test

^‡^ *P* value from Wilcoxon rank sum test

Regarding subjects in the lowest quartile of each indicator, ORs (95%CIs) for those in the highest quartile were 2.29 (1.78, 2.97), 2.65 (2.01, 3.48), 2.15 (1.73, 2.71), 4.58 (3.49, 6.02), 2.24 (1.81, 2.77) and 7.43 (5.75, 9.64) for NC, BF%, WHR, VAI, LAP, and RMR respectively after adjusting for all covariates (Table 2). The exposure–response trend of OSA with six indicators was observed (all *P*_-trend_ < 0.05). Every 1-unit increment of NC, BF%, and VAI was associated with a 13%, 9%, and 14% increased risk of OSA, respectively; every 0.01-unit increment of WHR was associated with a 3% increased risk of OSA; every 10-unit increment of LAP and RMR was associated with a 2% and 4% increased risk of OSA, respectively. The sensitivity analysis yielded consistent results that six indicators were positively associated with an increased OSA risk, and the positive association was independent of a woman's menopausal status (supplementary table S 1 and S2).

**Table 2:** Association between adiposity indicators and obstructive sleep apnea

Adiposity indicators	*N*		Effect		
	Non-OSA group	OSA group	Unadjusted OR (95% CI)	Adjusted OR (95% CI)^a^	Adjusted OR (95% CI)^b^
NC, cm					
Q1 (≤ 32.10)	2341	155	1.00	1.00	1.00
Q2 (> 32.10 ~ ≤ 34.20)	2197	309	2.12 (1.74, 2.60)	2.09 (1.71, 2.56)	1.40 (1.14, 1.73)
Q3 (> 34.20 ~ ≤ 36.60)	1929	396	3.10 (2.56, 3.78)	3.12 (2.55, 3.83)	1.61 (1.30, 2.01)
Q4 (> 36.60)	1640	766	7.05 (5.89, 8.50)	7.49 (6.06, 9.30)	2.29 (1.78, 2.97)
*P* for trend			< 0.001	0.553	< 0.001
Every 1-unit increment			1.25 (1.23, 1.27)	1.29 (1.26, 1.32)	1.13 (1.10, 1.16)
BF%, %					
Q1 (≤ 26.10)	2073	381	1.00	1.00	1.00
Q2 (> 26.10 ~ ≤ 31.00)	2041	390	1.04 (0.89, 1.21)	2.00 (1.69, 2.36)	1.26 (1.06, 1.50)
Q3 (> 31.00 ~ ≤ 35.10)	2125	340	0.87 (0.74, 1.02)	4.13 (3.31, 5.17)	1.97 (1.55, 2.51)
Q4 (> 35.10)	1868	515	1.50 (1.30, 1.74)	8.56 (6.74, 10.91)	2.65 (2.01, 3.48)
*P* for trend			< 0.001	< 0.001	0.029
Every 1-unit increment			1.03 (1.02, 1.04)	1.17 (1.15, 1.18)	1.09 (1.07, 1.10)
WHR					
Q1 (≤ 0.83)	2303	132	1.00	1.00	1.00
Q2 (> 0.83 ~ ≤ 0.88)	2101	331	2.75 (2.23, 3.40)	2.39 (1.94, 2.97)	1.68 (1.35, 2.09)
Q3 (> 0.88 ~ ≤ 0.93)	1952	482	4.31 (3.53, 5.29)	3.45 (2.81, 4.25)	1.93 (1.56, 2.41)
Q4 (> 0.93)	1751	681	6.79 (5.59, 8.29)	5.02 (4.10, 6.19)	2.15 (1.73, 2.71)
*P* for trend			< 0.001	< 0.001	< 0.001
Every 0.01-unit increment			1.08 (1.07, 1.09)	1.07 (1.06, 1.08)	1.03 (1.02, 1.04)
VAI					
Q1 (≤ 5.00)	2662	128	1.00	1.00	1.00
Q2 (> 5.00 ~ ≤ 8.00)	2430	317	2.71 (2.20, 3.37)	2.58 (2.09, 3.20)	1.91 (1.54, 2.40)
Q3 (> 8.00 ~ ≤ 11.00)	1660	381	4.77 (3.88, 5.90)	4.32 (3.49, 5.38)	2.54 (2.00, 3.24)
Q4 (> 11.00)	1355	800	12.28 (10.11, 15.02)	10.91 (8.85, 13.54)	4.58 (3.49, 6.02)
*P* for trend			< 0.001	0.302	< 0.001
Every 1-unit increment			1.21 (1.20, 1.23)	1.20 (1.19, 1.22)	1.14 (1.12, 1.16)
LAP					
Q1 (≤ 18.02)	2275	160	1.00	1.00	1.00
Q2 (> 18.02 ~ ≤ 31.12)	2099	333	2.26 (1.85, 2.75)	2.18 (1.79, 2.67)	1.43 (1.16, 1.76)
Q3 (> 31.12 ~ ≤ 51.60)	1967	466	3.37 (2.79, 4.08)	3.32 (2.74, 4.04)	1.79 (1.46, 2.21)
Q4 (> 51.60)	1766	667	5.37 (4.48, 6.47)	5.38 (4.47, 6.51)	2.24 (1.81, 2.77)
*P* for trend			< 0.001	< 0.001	< 0.001
Every 10-unit increment			1.07 (1.06, 1.09)	1.07 (1.06, 1.09)	1.02 (1.01, 1.03)
RMR, Kcal/day					
Q1 (≤ 1163.00)	2282	154	1.00	1.00	1.00
Q2 (> 1163.00 ~ ≤ 1285.00)	2163	282	1.93 (1.58, 2.38)	2.05 (1.67, 2.52)	1.82 (1.47, 2.24)
Q3 (> 1285.00 ~ ≤ 1467.00)	1969	451	3.39 (2.81, 4.13)	4.16 (3.40, 5.10)	3.31 (2.69, 4.09)
Q4 (> 1467.00)	1693	739	6.47 (5.39, 7.80)	11.19 (8.81, 14.28)	7.43 (5.75, 9.64)
*P* for trend			< 0.001	< 0.001	0.044
Every 10-unit increment			1.03 (1.02, 1.04)	1.04 (1.03, 1.05)	1.04 (1.03, 1.05)

*Abbreviation: NC* Neck circumference, *BF %* Body fat percentage, *WHR* Waist hip ratio, *VAI* Visceral adiposity index, *LAP* The lipid accumulation product, *RMR* The resting metabolic rate

^a^Adjustment for age, sex, marital status, education, smoking, alcohol drinking, fruit intake, vegetable intake, work intensity, and leisure-time physical activity

^b^Further adjustment for NC, WHR, BF%, VAI, LAP, and RMR

In stratified analyses by age, sex, and dyslipidemia, the associations of NC, BF%, WHR, VAI, LAP, and RMR with OSA were not significantly changed (Tables 3, 4 and 5). The associations of NC, BF%, WHR, VAI, and RMR with OSA risk were stronger in the middle-aged than in the elderly (*P*_-interaction_ < 0.001); the associations of VAI and RMR with OSA were slightly stronger in women than in men (*P*_-interaction_ = 0.020 and 0.024, respectively); and the associations of BF%, WHR, and LAP with OSA were stronger in the non-dyslipidemia group than in the dyslipidemia group (all *P*_-interaction_ < 0.05).

**Table 3:** Association between adiposity indicators and obstructive sleep apnea by age

Adiposity indicators	The middle-aged (35–64 years old)				The elderly (65 years old and above)			
	Non-OSA group	OSA group	Unadjusted OR (95% CI)	Adjusted OR (95% CI)^a^	Non-OSA group	OSA group	Unadjusted OR (95% CI)	Adjusted OR (95% CI)^a^
NC, cm								
Q1	1866	107	1.00	1.00	507	55	1.00	1.00
Q2	1631	194	2.07 (1.63, 2.66)	1.31 (1.02, 1.70)	444	103	2.14 (1.51, 3.05)	1.58 (1.10, 2.30)
Q3	1583	290	3.19 (2.54, 4.04)	1.62 (1.25, 2.10)	436	111	2.35 (1.67, 3.34)	1.45 (0.98, 2.16)
Q4	1284	577	7.84 (6.33, 9.79)	2.34 (1.72, 3.18)	356	189	4.89 (3.54, 6.85)	2.15 (1.36, 3.43)
*P* for trend			< 0.001	< 0.001			< 0.001	0.110
Every 1-unit increment			1.27 (1.24, 1.29)	1.14 (1.10, 1.18)			1.21 (1.17, 1.25)	1.13 (1.07, 1.20)
BF%, %								
Q1	1631	277	1.00	1.00	430	124	1.00	1.00
Q2	1622	272	0.99 (0.82, 1.18)	1.23 (1.01, 1.52)	446	114	0.89 (0.66, 1.18)	1.17 (0.84, 1.64)
Q3	1646	213	0.76 (0.63, 0.92)	2.02 (1.50, 2.71)	446	103	0.80 (0.60, 1.07)	1.74 (1.08, 2.81)
Q4	1465	406	1.63 (1.38, 1.93)	3.42 (2.47, 4.74)	421	117	0.96 (0.72, 1.28)	1.65 (0.98, 2.78)
*P* for trend			< 0.001	0.040			0.642	0.692
Every 1-unit increment			1.04 (1.03, 1.05)	1.10 (1.08, 1.12)			1.01 (0.99, 1.02)	1.05 (1.02, 1.09)
WHR								
Q1	1801	82	1.00	1.00	484	66	1.00	1.00
Q2	1665	218	2.88 (2.22, 3.76)	1.75 (1.34, 2.31)	423	127	2.20 (1.60, 3.06)	1.62 (1.16, 2.28)
Q3	1546	337	4.79 (3.75, 6.19)	2.07 (1.59, 2.72)	425	126	2.17 (1.58, 3.02)	1.38 (0.98, 1.96)
Q4	1352	531	8.63 (6.80, 11.07)	2.45 (1.86, 3.24)	411	139	2.48 (1.81, 3.44)	1.29 (0.90, 1.86)
*P* for trend			< 0.001	< 0.001			< 0.001	0.256
Every 0.01-unit increment			1.10 (1.09, 1.11)	1.04 (1.02, 1.05)			1.04 (1.03, 1.06)	1.01 (0.99, 1.03)
VAI								
Q1	2262	90	1.00	1.00	556	58	1.00	1.00
Q2	1399	155	2.78 (2.13, 3.65)	2.01 (1.53, 2.65)	499	110	2.11 (1.51, 2.99)	1.58 (1.10, 2.29)
Q3	1766	349	4.97 (3.93, 6.35)	2.68 (2.05, 3.53)	357	110	2.95 (2.10, 4.19)	1.79 (1.20, 2.69)
Q4	937	574	15.4 (12.24, 19.58)	5.46 (3.97, 7.57)	331	180	5.21 (3.79, 7.27)	2.34 (1.46, 3.77)
*P* for trend			< 0.001	< 0.001			< 0.001	0.570
Every 1-unit increment			1.24 (1.22, 1.26)	1.17 (1.14, 1.20)			1.14 (1.12, 1.17)	1.10 (1.06, 1.14)
LAP								
Q1	1791	96	1.00	1.00	474	78	1.00	1.00
Q2	1650	229	2.59 (2.03, 3.33)	1.61 (1.25, 2.09)	450	99	1.34 (0.97, 1.85)	1.06 (0.75, 1.50)
Q3	1553	330	3.96 (3.14, 5.05)	1.97 (1.53, 2.56)	420	129	1.87 (1.37, 2.55)	1.36 (0.96, 1.93)
Q4	1370	513	6.99 (5.58, 8.83)	2.61 (2.01, 3.40)	399	152	2.32 (1.71, 3.15)	1.54 (1.07, 2.22)
*P* for trend			< 0.001	< 0.001			< 0.001	0.358
Every 10-unit increment			1.09 (1.07, 1.10)	1.03 (1.01, 1.04)			1.04 (1.02, 1.06)	1.01 (1.00, 1.04)
RMR, Kcal/day								
Q1	1801	89	1.00	1.00	498	58	1.00	1.00
Q2	1693	191	2.28 (1.77, 2.97)	2.02 (1.55, 2.64)	456	89	1.68 (1.18, 2.40)	1.58 (1.11, 2.28)
Q3	1544	331	4.34 (3.42, 5.56)	4.00 (3.10, 5.22)	424	126	2.55 (1.83, 3.59)	2.48 (1.71, 3.62)
Q4	1326	557	8.50 (6.76, 10.81)	9.26 (6.77, 12.75)	365	185	4.35 (3.16, 6.06)	4.57 (2.86, 7.33)
*P* for trend			< 0.001	0.024			< 0.001	0.807
Every 10-unitincrement			1.04 (1.03, 1.05)	1.05 (1.04, 1.06)			1.03 (1.02, 1.04)	1.04 (1.03, 1.05)

*Abbreviation: NC* Neck circumference, *BF %* Body fat percentage, *WHR* Waist hip ratio, *VAI* Visceral adiposity index, *LAP* The lipid accumulation product, *RMR* The resting metabolic rate

^a^Adjustment for age, sex, marital status, education, smoking, alcohol drinking, fruit intake, vegetable intake, work intensity, leisure-time physical activity, NC, WHR, BF%, VAI, LAP, and RMR

**Table 4:** Association between adiposity indicators and obstructive sleep apnea by sex

Adiposity indicators	Male				Female			
	Non-OSA group	OSA group	Unadjusted OR (95% CI)	Adjusted OR (95% CI)^a^	Non-OSA group	OSA group	Unadjusted OR (95% CI)	Adjusted OR (95% CI)^a^
NC, cm								
Q1	723	110	1.00	1.00	1628	77	1.00	1.00
Q2	676	138	1.34 (1.02, 1.76)	1.05 (0.79, 1.40)	1625	199	2.59 (1.98, 3.42)	1.78 (1.35, 2.37)
Q3	639	238	2.45 (1.91, 3.15)	1.55 (1.17, 2.05)	1149	180	3.31 (2.52, 4.39)	1.73 (1.29, 2.34)
Q4	431	318	4.85 (3.80, 6.23)	2.28 (1.66, 3.13)	1236	366	6.26 (4.87, 8.14)	2.24 (1.66, 3.05)
*P* for trend			< 0.001	0.852			< 0.001	0.200
Every 1-unit increment			1.26 (1.22, 1.30)	1.14 (1.10, 1.19)			1.29 (1.26, 1.33)	1.12 (1.08, 1.17)
BF%, %								
Q1	739	90	1.00	1.00	1567	56	1.00	1.00
Q2	645	172	2.19 (1.67, 2.89)	1.56 (1.18, 2.08)	1482	139	2.62 (1.92, 3.63)	1.80 (1.31, 2.51)
Q3	587	227	3.18 (2.44, 4.16)	1.82 (1.36, 2.43)	1362	243	4.99 (3.73, 6.80)	2.41 (1.76, 3.35)
Q4	498	315	5.19 (4.02, 6.77)	2.29 (1.69, 3.11)	1227	384	8.76 (6.61, 11.82)	2.88 (2.06, 4.06)
*P* for trend			< 0.001	0.893			< 0.001	0.212
Every 1-unit increment			1.15 (1.12, 1.17)	1.08 (1.05, 1.10)			1.2 (1.18, 1.22)	2.45 (1.90, 3.18)
WHR								
Q1	720	98	1.00	1.00	1551	64	1.00	1.00
Q2	664	150	1.66 (1.26, 2.19)	1.14 (0.86, 1.52)	1444	170	2.85 (2.13, 3.86)	1.76 (1.30, 2.40)
Q3	583	240	3.02 (2.34, 3.93)	1.74 (1.32, 2.31)	1333	283	5.15 (3.91, 6.87)	2.45 (1.83, 3.32)
Q4	502	316	4.62 (3.60, 5.98)	2.02 (1.51, 2.71)	1310	305	5.64 (4.3, 7.52)	1.97 (1.45, 2.71)
*P* for trend			< 0.001	0.575			< 0.001	0.118
Every 0.01-unit increment			1.09 (1.07, 1.10)	1.04 (1.02, 1.05)			1.07 (1.06, 1.08)	1.09 (1.07, 1.12)
VAI								
Q1	903	101	1.00	1.00	1612	50	1.00	1.00
Q2	675	185	2.45 (1.89, 3.19)	1.76 (1.33, 2.34)	1446	132	2.94 (2.12, 4.14)	2.07 (1.48, 2.93)
Q3	530	210	3.54 (2.74, 4.61)	2.20 (1.62, 2.99)	1549	244	5.08 (3.75, 7.01)	2.69 (1.93, 3.82)
Q4	361	308	7.63 (5.93, 9.89)	3.49 (2.44, 5.01)	1031	396	12.38 (9.22, 16.98)	4.37 (2.99, 6.47)
*P* for trend			< 0.001	0.411			< 0.001	0.053
Every 1-unit increment			1.18 (1.16, 1.21)	1.14 (1.10, 1.17)			1.23 (1.20, 1.25)	1.15 (1.11, 1.18)
LAP								
Q1	728	91	1.00	1.00	1548	67	1.00	1.00
Q2	648	169	2.09 (1.59, 2.76)	1.36 (1.02, 1.83)	1453	162	2.58 (1.93, 3.47)	1.52 (1.12, 2.07)
Q3	581	238	3.28 (2.52, 4.29)	1.82 (1.37, 2.44)	1383	232	3.88 (2.94, 5.17)	1.78 (1.33, 2.42)
Q4	512	306	4.78 (3.70, 6.23)	2.23 (1.65, 3.03)	1254	361	6.65 (5.11, 8.79)	2.27 (1.67, 3.10)
*P* for trend			< 0.001	0.751			< 0.001	0.263
Every 10-unit increment			1.07 (1.05, 1.09)	1.02 (1.01, 1.04)			1.08 (1.06, 1.10)	1.02 (1.01, 1.04)
RMR, Kcal/day								
Q1	705	118	1.00	1.00	1523	96	1.00	1.00
Q2	670	145	1.29 (0.99, 1.69)	1.26 (0.96, 1.65)	1508	120	1.26 (0.96, 1.67)	1.25 (0.95, 1.66)
Q3	596	224	2.25 (1.76, 2.88)	2.21 (1.70, 2.89)	1378	224	2.58 (2.02, 3.32)	2.45 (1.90, 3.18)
Q4	498	317	3.80 (3.00, 4.85)	3.80 (2.88, 5.03)	1229	382	4.93 (3.91, 6.27)	4.45 (3.46, 5.77)
*P* for trend			< 0.001	0.665			< 0.001	0.343
Every 10-unit increment			1.04 (1.03, 1.05)	1.04 (1.03, 1.05)			1.05 (1.05, 1.06)	1.05 (1.04, 1.06)

*Abbreviation: NC* Neck circumference, *BF %* Body fat percentage, *WHR* Waist hip ratio, *VAI* Visceral adiposity index, *LAP* The lipid accumulation product, *RMR* The resting metabolic rate

^a^ djustment for age, marital status, education, smoking, alcohol drinking, fruit intake, vegetables intake, work intensity, leisure-time physical activity, NC, WHR, BF%, VAI, LAP, and RMR

**Table 5:** Association between adiposity indicators and obstructive sleep apnea by history of dyslipidemia

Adiposity indicators	Non-dyslipidemia				Dyslipidemia			
	Non-OSA group	OSA group	Unadjusted OR (95% CI)	Adjusted OR (95% CI)^a^	Non-OSA group	OSA group	Unadjusted OR (95% CI)	Adjusted OR (95% CI)^a^
NC, cm								
Q1	808	35	1.00	1.00	1606	131	1.00	1.00
Q2	639	78	2.82 (1.88, 4.30)	1.71 (1.12, 2.66)	1492	227	1.87 (1.49, 2.34)	1.29 (1.02, 1.64)
Q3	528	103	4.50 (3.05, 6.79)	2.26 (1.46, 3.56)	1441	291	2.48 (2.00, 3.09)	1.33 (1.04, 1.70)
Q4	522	198	8.76 (6.09, 12.94)	2.87 (1.73, 4.82)	1071	563	6.44 (5.27, 7.94)	2.18 (1.63, 2.93)
*P* for trend			< 0.001	< 0.001			< 0.001	< 0.001
Every 1-unit increment			1.28 (1.23, 1.32)	1.15 (1.08, 1.22)			1.24 (1.22, 1.27)	1.12 (1.09, 1.16)
BF%, %								
Q1	646	83	1.00	1.00	1410	304	1.00	1.00
Q2	654	85	1.01 (0.73, 1.40)	1.09 (0.75, 1.57)	1408	312	1.03 (0.86, 1.22)	1.37 (1.12, 1.67)
Q3	636	79	0.97 (0.70, 1.34)	1.83 (1.14, 2.97)	1479	240	0.75 (0.63, 0.90)	1.84 (1.39, 2.45)
Q4	561	167	2.32 (1.74, 3.10)	3.32 (1.92, 5.76)	1313	356	1.26 (1.06, 1.49)	2.44 (1.78, 3.36)
*P* for trend			< 0.001	0.701			0.124	0.040
Every 1-unit increment			1.06 (1.04, 1.08)	1.08 (1.04, 1.11)			1.02 (1.01, 1.03)	1.09 (1.06, 1.11)
WHR								
Q1	706	22	1.00	1.00	1587	117	1.00	1.00
Q2	653	74	3.64 (2.27, 6.05)	2.00 (1.23, 3.38)	1456	251	2.34 (1.86, 2.95)	1.51 (1.19, 1.93)
Q3	594	134	7.24 (4.65, 11.81)	2.74 (1.71, 4.59)	1345	360	3.63 (2.92, 4.54)	1.75 (1.39, 2.23)
Q4	544	184	10.85 (7.03, 17.58)	2.58 (1.57, 4.39)	1222	484	5.37 (4.35, 6.69)	1.92 (1.50, 2.47)
P for trend			< 0.001	< 0.001			< 0.001	0.010
Every 0.01-unit increment			1.10 (1.09, 1.12)	1.04 (1.02, 1.06)			1.08 (1.07, 1.09)	1.02 (1.01, 1.03)
VAI								
Q1	981	30	1.00	1.00	1681	98	1.00	1.00
Q2	521	66	4.14 (2.68, 6.54)	2.60 (1.65, 4.17)	1702	217	2.19 (1.71, 2.81)	1.63 (1.26, 2.11)
Q3	540	100	6.06 (4.02, 9.37)	2.83 (1.78, 4.58)	1212	287	4.06 (3.20, 5.19)	2.35 (1.79, 3.11)
Q4	455	218	15.67 (10.7, 23.75)	4.38 (2.59, 7.56)	1015	610	10.31 (8.25, 13.00)	4.27 (3.13, 5.85)
*P* for trend			< 0.001	< 0.001			< 0.001	0.066
Every 1-unit increment			1.23 (1.20, 1.26)	1.14 (1.09, 1.19)			1.20 (1.18, 1.22)	1.14 (1.11, 1.17)
LAP								
Q1	702	26	1.00	1.00	1560	146	1.00	1.00
Q2	655	73	3.01 (1.92, 4.85)	1.76 (1.10, 2.89)	1461	244	1.78 (1.44, 2.22)	1.24 (0.98, 1.56)
Q3	607	121	5.38 (3.53, 8.50)	2.34 (1.47, 3.83)	1353	352	2.78 (2.27, 3.42)	1.60 (1.28, 2.01)
Q4	533	194	9.83 (6.55, 15.35)	3.15 (1.95, 5.23)	1236	470	4.06 (3.33, 4.98)	1.86 (1.48, 2.36)
*P* for trend			< 0.001	0.001			< 0.001	0.015
Every 10-unit increment			1.46 (1.38, 1.55)	1.19 (1.10, 1.28)			1.06 (1.05, 1.07)	1.02 (1.01, 1.03)
RMR, Kcal/day								
Q1	698	31	1.00	1.00	1595	120	1.00	1.00
Q2	653	74	2.55 (1.67, 3.98)	1.83 (1.18, 2.91)	1485	219	1.96 (1.55, 2.48)	1.86 (1.47, 2.36)
Q3	606	122	4.53 (3.05, 6.93)	2.68 (1.70, 4.32)	1381	317	3.05 (2.45, 3.82)	2.99 (2.35, 3.81)
Q4	540	187	7.80 (5.32, 11.79)	4.10 (2.30, 7.41)	1149	556	6.43 (5.22, 7.98)	7.26 (5.39, 9.83)
*P* for trend			< 0.001	0.830			< 0.001	0.703
Every 10-unit increment			1.03 (1.03, 1.04)	1.03 (1.02, 1.04)			1.03 (1.03, 1.04)	1.04 (1.04, 1.05)

*Abbreviation: NC* Neck circumference, *BF %* Body fat percentage, *WHR* Waist hip ratio, *VAI* Visceral adiposity index, *LAP* The lipid accumulation product, *RMR* The resting metabolic rate

^a^Adjustment for age, sex, marital status, education, smoking, alcohol drinking, fruit intake, vegetables intake, work intensity, leisure-time physical activity, NC, WHR, BF%, VAI, LAP, and RMR

## Discussion

To our knowledge, this is the first study to comprehensively examine the effects of common and novel adiposity indicators on the risk of OSA. This large population-based study found that NC, BF%, WHR, VAI, LAP, and RMR were all independently and positively associated with the OSA risk. The stratified and sensitivity analysis yielded similar results, indicating the robustness of the results.

This study found that the OSA risk increased with NC increment, which was consistent with previous studies [13, 29]. Increased NC implies more adipose tissue adjacent to the upper airway, with consequent reduced upper airway caliber and predisposes to OSA [29]. By contrast, BF% has received little attention in the etiology of OSA. We found that every 1-unit increment of BF% was associated with a 9% increased risk of OSA, indicating excessive fat accumulation was a risk factor for OSA regardless of fat distribution.-T-he risk of tissue hypoxia develops as adipocyte hypertrophy continues, with subsequent inflammatory activation, oxidative stress, and increased sympathetic activity, which eventually leads to the occurrence of OSA [7].

Indicators of abdominal adiposity including WHR, VAI, and LAP were all found to be independent risk factors for OSA, which was consistent with previous studies [13, 30]. Two separate observational and longitudinal studies concluded that abdominal obesity characterized by WC and HC was more strongly correlated with OSA than general obesity in China [11]. A cross-sectional study suggested that VAI was significantly associated with OSA risk, with all significantly correlated with an apnea–hypopnea index (AHI), and mean and lowest oxygen saturation [31]. LAP was initially developed for recognizing cardiovascular risk and then applied in the identification of metabolic diseases and OSA. Zou et al. suggested that LAP might be one key exponent in screening for OSA [13]. Abdominal adiposity accumulation may reduce pharyngeal lumen size, decrease upper airway muscle protective force and size, and affect restrictive respiratory dysfunction, finally leading to daytime hypoxemia and the development of OSA [30, 32]. RMR was positively associated with the OSA risk in this study. A university-based cross-sectional study showed that increased resting energy expenditure was independently associated with AHI, resulting in greater severity of sleep-disordered breathing [33]. Another study conducted a three-month continuous positive airway pressure therapy for OSA patients and found that the basal metabolic rate (equal to the RMR) was reduced in the absence of changes in physical activity, thus favoring a positive energy balance in terms of energy expenditure [34].

The stratified analysis by age showed that the associations of NC, BF%, WHR, VAI, LAP, and RMR with OSA risk were stronger in the middle-aged than in the elderly. This disparity could be explained by the contradictory effect of adipose tissue distribution on the elderly. Many elderly obese may exhibit late-onset obesity, health risks, and comorbidities not manifest due to its short duration [35]. Besides, Tung and colleagues followed 4,000 older adults for 5 years and found that older men were resistant to hazards of overweight and adiposity; mild-grade overweight or obesity might be protective [36]. The aging process is indeed characterized by an increase in total body fat mass and a concomitant decrease in lean mass and bone density, independent of general and physiological fluctuations in weight and BMI [37]. A systematic review concluded that five-year increases in the visceral adipose tissue (VAT) area declined with the advanced age group in both men and women, regardless of race [38].

In the stratified analysis by sex, the negative associations of VAI and RMR with OSA were stronger in women than in men. Studies have shown that women tend to have higher percent body fat throughout the entire life span with relatively more adipose tissue deposited in the hips and thighs, while men tend to have a greater degree of visceral obesity with excess fat more concentrated in the abdomen and neck [32]. These yielded consistent results that women had higher BF% (33.6% vs. 24.7%), lower VAI (6 vs. 11), and lower WHR (0.86 vs. 0.91) than men. However, it is reported that menopause is followed by redistribution of adipose tissue towards a more central phenotype and raised visceral adiposity in women during the peri-menopausal transition presumably due to the fall in estrogen levels [32, 39]. 67.3% of the women in this study were menopausal. Sensitivity analysis showed that the association between adiposity indicators and OSA was independent of menopausal status, which indicated that even premenopausal women should pay more attention to OSA prevention. Moreover, the energy expenditure in women was lower than in men, and women were more susceptible to accumulating fat tissue, especially old-age women.

In addition, among non-dyslipidemia subjects, BF%, WHR, LAP, and RMR were more strongly associated with OSA risk than those with dyslipidemia. There are complex interactions between obesity, dyslipidemia, and OSA, and in many cases, they coexist. Studies have reported that dyslipidemia predisposes to excess fatty deposition in the neck, thorax, and abdomen, impacts the pulmonary system and thereby increases OSA susceptibility [40]. Participants not suffering from dyslipidemia may be more sensitive to visceral fat accumulation, leading to a higher risk of OSA, compared to those with dyslipidemia.

## Study strengths and limitations

There are some strengths. First, the multi-stage sampling method was applied to recruit participants from the general population in Guangzhou communities, which greatly reduced the selection bias and enhanced the representativeness of the sample. Second, the large sample size improved the statistical power and allowed for comparisons by age, sex, and history of dyslipidemia. Third, this study considered the effect of regional fat distribution on OSA and combined traditional and novel parameters of adiposity. Finally, we performed several stratified and sensitivity analyses and the results of which showed consistent associations, indicating the robustness of our results to a certain degree.

Some limitations also exist. First, the cross-sectional design could not provide causal inference according to our report. However, the dose–response relationship between adiposity indicators and OSA enhanced the existence of causation. Second, OSA was determined by the Berlin Questionnaire due to the lack of polysomnography during data collection, which is a commonly used validated tool in epidemiological and clinical research [24]. Compared with many other screening questionnaires that are lengthy and complicated, the Berlin questionnaire has been widely adopted and validated in various populations because of its ease of use, efficiency, and good sensitivity. Third, adiposity indicators were measured by Omron body composition monitor, which may not provide measurements as accurate as other advanced methods, such as Dual-energy X-ray absorptiometry [41]. The accuracy of the measurements was susceptible to being affected by body temperature, food ingestion, ambient temperature, and humidity. However, the portable Omron device has been applied in several studies and could provide a rapid, non-invasive, and reasonably accurate measurement of body composition [42–44]. But considering the cost and convenience, it was more practical to use portable protocols in this large-scale population study.

## Conclusion

NC, BF%, WHR, VAI, LAP, and RMR were all independently and positively associated with OSA risk, regardless of age, sex, history of dyslipidemia, and menopausal status. Application of these new indicators could help to more comprehensively reflect and predict the risk of OSA in the general population. More attention should be paid to the middle-aged, women, or non-dyslipidemia population.

### Supplementary Information

**Additional file 1: Supplementary table S1.** Sensitivity analysis on the association between adjusted adiposity indicators and obstructive sleep apnea. **Supplementary table S2.** Association between adiposity indicators and obstructive sleep apnea by menopause status among women.

## Data Availability

The data used to support the findings of this study are available from the corresponding author upon request. A proposal with a detailed description of study objectives and a statistical analysis plan will be needed for the evaluation of the reasonability of requests if someone requests data sharing.

## Abbreviations

AHI: Apnea–hypopnea index
BF%: Body fat percentage
BMI: Body mass index
BQ: Berlin Questionnaire
CI: Confidence interval
IL: Interleukin
IQR: Interquartile range
LAP: Lipid accumulation product
LTPA: Leisure-time physical activity
NC: Neck circumference
OR: Odds ratio
OSA: Obstructive sleep apnea
RMR: Resting metabolic rate
SD: Standard deviation
VAI: Visceral adiposity index
VAT: Visceral adipose tissue
VIF: Variance inflation factors
WC: Waist circumference
WHR: Waist-to-hip ratio
