# Supplementary material for: Association of adiposity with risk of obstructive sleep apnea: a population-based study
Source: BMC Public Health. 2023 Sep 21;23:1835. doi: 10.1186/s12889-023-16695-4 (PMC10512644; doi:10.1186/s12889-023-16695-4)
Supplement: Supplementary file 1 — Additional file 1: Supplementary table S1. Sensitivity analysis on the association between adjusted adiposity indicators and obstructive sleep apnea. Supplementary table S2. Association between adiposity indicators and obstructive sleep apnea by menopause status among women. [file 12889_2023_16695_MOESM1_ESM.docx]

**Supplementary tables**

Supplementary table S1. Sensitivity analysis on the association between adjusted adiposity indicators and obstructive sleep apnea

| Adiposity indicators | N | |  | Effect | | |
| --- | --- | --- | --- | --- | --- | --- |
|  | Non-OSA group | OSA group |  | Unadjusted OR (95% CI) | Adjusted OR (95% CI) ^a^ | Adjusted OR (95% CI) ^b^ |
| NC, cm |  |  |  |  |  |  |
| Q1 (≤32.30) | 2331 | 168 |  | 1.00 | 1.00 | 1.00 |
| Q2 (> 32.30 ~ ≤34.30) | 2089 | 300 |  | 1.99 (1.64, 2.43) | 1.91 (1.57, 2.34) | 1.30 (1.06, 1.59) |
| Q3 (> 34.30~ ≤36.30) | 1990 | 476 |  | 3.32 (2.76, 4.01) | 3.07 (2.53, 3.74) | 1.53 (1.24, 1.89) |
| Q4 (> 36.30) | 1697 | 682 |  | 5.58 (4.67, 6.69) | 4.94 (4.02, 6.08) | 1.75 (1.39, 2.21) |
| *P* for trend |  |  |  | <0.001 | 0.039 | <0.001 |
| Every 1-unit increment |  |  |  | 1.24 (1.22, 1.27) | 1.23 (1.21, 1.27) | 1.07 (1.04, 1.10) |
| BF%, % |  |  |  |  |  |  |
| Q1 (≤ 26.70) | 2049 | 417 |  | 1.00 | 1.00 | 1.00 |
| Q2 (> 26.70 ~ ≤31.00) | 2215 | 440 |  | 0.98 (0.84, 1.13) | 1.63 (1.39, 1.91) | 1.17 (0.99, 1.39) |
| Q3 (> 31.00 ~ ≤34.60) | 1898 | 284 |  | 0.74 (0.62, 0.87) | 2.25 (1.82, 2.78) | 1.37 (1.10, 1.71) |
| Q4 (> 34.60) | 1945 | 485 |  | 1.23 (1.06, 1.42) | 4.09 (3.30, 5.09) | 1.75 (1.39, 2.22) |
| *P* for trend |  |  |  | 0.079 | <0.001 | 0.047 |
| Every 1-unit increment |  |  |  | 1.02 (1.01, 1.03) | 1.12 (1.10, 1.14) | 1.05 (1.04, 1.07) |
| WHR |  |  |  |  |  |  |
| Q1 (≤ 0.84) | 2268 | 165 |  | 1.00 | 1.00 | 1.00 |
| Q2 (> 0.84 ~ ≤ 0.88) | 1601 | 274 |  | 2.35 (1.92, 2.89) | 2.08 (1.69, 2.56) | 1.58 (1.28, 1.95) |
| Q3 (> 0.88 ~ ≤ 0.92) | 2448 | 550 |  | 3.09 (2.58, 3.72) | 2.57 (2.13, 3.10) | 1.60 (1.32, 1.95) |
| Q4 (> 0.92) | 1790 | 637 |  | 4.89 (4.09, 5.88) | 3.62 (3.00, 4.38) | 1.84 (1.51, 2.26) |
| *P* for trend |  |  |  | <0.001 | <0.001 | <0.001 |
| Every 0.01-unit increment |  |  |  | 1.11 (1.10, 1.13) | 1.09 (1.08, 1.11) | 1.04 (1.03, 1.06) |
| VAI |  |  |  |  |  |  |
| Q1 (≤ 5.00) | 2527 | 124 |  | 1.00 | 1.00 | 1.00 |
| Q2 (> 5.00 ~ ≤ 8.00) | 2430 | 317 |  | 2.66 (2.15, 3.31) | 2.48 (2.00, 3.09) | 1.75 (1.40, 2.19) |
| Q3 (> 8.00 ~ ≤ 11.00) | 1876 | 509 |  | 5.53 (4.52, 6.81) | 4.81 (3.91, 5.96) | 2.39 (1.89, 3.03) |
| Q4 (> 11.00) | 1274 | 676 |  | 10.81 (8.86, 13.30) | 9.00 (7.27, 11.21) | 3.38 (2.62, 4.38) |
| *P* for trend |  |  |  | <0.001 | 0.022 | <0.001 |
| Every 1-unit increment |  |  |  | 1.22 (1.20, 1.23) | 1.20 (1.18, 1.21) | 1.09 (1.07, 1.11) |
| LAP |  |  |  |  |  |  |
| Q1 (≤ 19.21) | 2253 | 181 |  | 1.00 | 1.00 | 1.00 |
| Q2 (> 19.21 ~ ≤ 32.67) | 2091 | 342 |  | 2.04 (1.69, 2.47) | 1.95 (1.61, 2.37) | 1.39 (1.14, 1.70) |
| Q3 (> 32.67 ~ ≤ 48.55) | 1980 | 452 |  | 2.84 (2.37, 3.42) | 2.75 (2.28, 3.31) | 1.58 (1.30, 1.94) |
| Q4 (> 48.55) | 1783 | 651 |  | 4.54 (3.82, 5.43) | 4.52 (3.78, 5.42) | 2.21 (1.81, 2.69) |
| *P* for trend |  |  |  | <0.001 | <0.001 | 0.001 |
| Every 10-unit increment |  |  |  | 1.18 (1.16, 1.21) | 1.19 (1.16, 1.21) | 1.09 (1.07, 1.12) |
| RMR, Kcal/day |  |  |  |  |  |  |
| Q1 (≤ 1175.00) | 2286 | 160 |  | 1.00 | 1.00 | 1.00 |
| Q2 (> 1175.00 ~ ≤1299.00) | 2123 | 304 |  | 2.05 (1.68, 2.50) | 2.07 (1.69, 2.54) | 1.97 (1.61, 2.42) |
| Q3 (> 1299.00 ~ ≤1444.00) | 1927 | 504 |  | 3.74 (3.10, 4.52) | 3.77 (3.10, 4.61) | 3.33 (2.72, 4.09) |
| Q4 (> 1444.00) | 1771 | 658 |  | 5.31 (4.43, 6.40) | 5.60 (4.46, 7.05) | 7.01 (5.57, 8.86) |
| *P* for trend |  |  |  | <0.001 | 0.037 | <0.001 |
| Every 10-unit increment |  |  |  | 1.03 (1.02, 1.04) | 1.04 (1.03, 1.05) | 1.04 (1.03, 1.05) |

*Abbreviation:* *NC*, neck circumference; *BF%*, body fat percentage; *WHR*, waist hip ratio; *VAI*, visceral adiposity index; *LAP*, lipid accumulation product; *RMR*, resting metabolic rate. The analysis was conducted by adjusting the upper and lower 2.5% of each adiposity indicator to the means.

^a^ Adjustment for age, sex, marital status, education, smoking, alcohol drinking, fruit intake, vegetable intake, work intensity, and leisure-time physical activity.

^b^ Further adjustment for NC, WHR, BF%, VAI, LAP, and RMR.

Supplementary table S2. Association between adiposity indicators and obstructive sleep apnea by menopause status among women

| Adiposity indicators | Premenopausal women (N=2,125) | | | |  | Menopausal women (N=4,335) | | | |
| --- | --- | --- | --- | --- | --- | --- | --- | --- | --- |
|  | Non-OSA  group | OSA  group | Unadjusted OR  (95% CI) | Adjusted OR  (95% CI) ^a^ |  | Non-OSA  group | OSA  group | Unadjusted OR  (95% CI) | Adjusted OR  (95% CI) ^a^ |
| NC, cm |  |  |  |  |  |  |  |  |  |
| Q1 | 529 | 4 | 1.00 | 1.00 |  | 1058 | 70 | 1.00 | 1.00 |
| Q2 | 524 | 35 | 8.83 (3.50, 29.72) | 5.92 (2.26, 20.52) |  | 1037 | 163 | 2.38 (1.78, 3.20) | 1.68 (1.25, 2.29) |
| Q3 | 460 | 44 | 12.65 (5.08, 42.25) | 5.43 (2.08, 18.74) |  | 789 | 141 | 2.70 (2.01, 3.67) | 1.55 (1.12, 2.16) |
| Q4 | 432 | 97 | 29.7 (12.31, 97.58) | 6.88 (2.61, 23.81) |  | 809 | 268 | 5.01 (3.81, 6.66) | 2.05 (1.47, 2.88) |
| *P* for trend |  |  | <0.001 | 0.581 |  |  |  | <0.001 | 0.233 |
| Every 1-unit increment |  |  | 1.41 (1.33, 1.50) | 1.16 (1.06, 1.26) |  |  |  | 1.26 (1.22, 1.30) | 1.11 (1.06, 1.16) |
| BF%, % |  |  |  |  |  |  |  |  |  |
| Q1 | 549 | 7 | 1.00 | 1.00 |  | 1018 | 74 | 1.00 | 1.00 |
| Q2 | 494 | 22 | 3.49 (1.55, 8.90) | 2.47 (1.07, 6.45) |  | 970 | 130 | 1.84 (1.37, 2.49) | 1.34 (0.99, 1.83) |
| Q3 | 487 | 42 | 6.76 (3.21, 16.60) | 3.37 (1.54, 8.49) |  | 909 | 170 | 2.57 (1.94, 3.45) | 1.45 (1.07, 1.99) |
| Q4 | 415 | 109 | 20.6 (10.21, 49.22) | 4.82 (2.19, 12.18) |  | 796 | 268 | 4.63 (3.54, 6.13) | 1.99 (1.44, 2.76) |
| *P* for trend |  |  | <0.001 | 0.62 |  |  |  | <0.001 | 0.263 |
| Every 1-unit increment |  |  | 1.32 (1.26, 1.38) | 1.14 (1.07, 1.21) |  |  |  | 1.16 (1.14, 1.19) | 1.08 (1.05, 1.11) |
| WHR |  |  |  |  |  |  |  |  |  |
| Q1 | 525 | 7 | 1.00 | 1.00 |  | 1009 | 75 | 1.00 | 1.00 |
| Q2 | 503 | 28 | 4.17 (1.91, 10.46) | 2.64 (1.17, 6.80) |  | 926 | 159 | 2.31 (1.74, 3.10) | 1.59 (1.18, 2.15) |
| Q3 | 478 | 51 | 8.00 (3.84, 19.49) | 3.71 (1.71, 9.30) |  | 883 | 199 | 3.03 (2.30, 4.03) | 1.76 (1.31, 2.38) |
| Q4 | 439 | 94 | 16.06 (7.92, 38.47) | 4.79 (2.23, 11.93) |  | 875 | 209 | 3.21 (2.44, 4.27) | 1.53 (1.12, 2.09) |
| *P* for trend |  |  | 4.17 (1.91, 10.46) | 2.64 (1.17, 6.80) |  |  |  | <0.001 | 0.176 |
| Every 0.01-unit increment |  |  | 1.10 (1.08, 1.13) | 1.05 (1.02, 1.08) |  |  |  | 1.05 (1.04, 1.06) | 1.01 (1.00, 1.03) |
| VAI |  |  |  |  |  |  |  |  |  |
| Q1 | 771 | 9 | 1.00 | 1.00 |  | 1278 | 84 | 1.00 | 1.00 |
| Q2 | 284 | 13 | 3.92 (1.67, 9.60) | 2.65 (1.11, 6.59) |  | 904 | 128 | 2.15 (1.62, 2.88) | 1.66 (1.23, 2.25) |
| Q3 | 592 | 52 | 7.52 (3.87, 16.46) | 3.86 (1.91, 8.68) |  | 948 | 172 | 2.76 (2.11, 3.65) | 1.77 (1.29, 2.45) |
| Q4 | 298 | 106 | 30.47 (16.1, 65.49) | 8.57 (3.94, 20.51) |  | 563 | 258 | 6.97 (5.37, 9.14) | 3.40 (2.34, 4.97) |
| *P* for trend |  |  | <0.001 | 0.300 |  |  |  | <0.001 | 0.068 |
| Every 1-unit increment |  |  | 1.36 (1.30, 1.42) | 1.21 (1.13, 1.30) |  |  |  | 1.19 (1.16, 1.21) | 1.14 (1.10, 1.18) |
| LAP |  |  |  |  |  |  |  |  |  |
| Q1 | 527 | 3 | 1.00 | 1.00 |  | 1002 | 82 | 1.00 | 1.00 |
| Q2 | 504 | 29 | 10.11 (3.56, 42.40) | 5.58 (1.94, 23.63) |  | 949 | 134 | 1.73 (1.30, 2.31) | 1.15 (0.85, 1.56) |
| Q3 | 491 | 40 | 14.31 (5.16, 59.42) | 5.33 (1.86, 22.49) |  | 907 | 177 | 2.38 (1.81, 3.16) | 1.31 (0.98, 1.78) |
| Q4 | 423 | 108 | 44.85 (16.76, 183.1) | 11.46 (4.05, 48.11) |  | 835 | 249 | 3.64 (2.81, 4.78) | 1.61 (1.18, 2.20) |
| *P* for trend |  |  | <0.001 | 0.809 |  |  |  | <0.001 | 0.325 |
| Every 10-unit increment |  |  | 1.13 (1.09, 1.17) | 1.05 (1.01, 1.08) |  |  |  | 1.06 (1.04, 1.08) | 1.02 (1.01, 1.04) |
| RMR, Kcal/day |  |  |  |  |  |  |  |  |  |
| Q1 | 528 | 10 | 1.00 | 1.00 |  | 1006 | 86 | 1.00 | 1.00 |
| Q2 | 512 | 16 | 1.65 (0.75, 3.80) | 1.31 (0.58, 3.05) |  | 987 | 103 | 1.22 (0.91, 1.65) | 1.21 (0.89, 1.64) |
| Q3 | 486 | 42 | 4.56 (2.36, 9.72) | 3.37 (1.71, 7.28) |  | 898 | 174 | 2.27 (1.73, 2.99) | 2.11 (1.59, 2.80) |
| Q4 | 419 | 112 | 14.11 (7.67, 29.09) | 9.08 (4.77, 19.16) |  | 802 | 279 | 4.07 (3.15, 5.30) | 3.62 (2.74, 4.81) |
| *P* for trend |  |  | <0.001 | 0.595 |  |  |  | <0.001 | 0.398 |
| Every 10-unit increment |  |  | 1.07 (1.06, 1.09) | 1.06 (1.05, 1.08) |  |  |  | 1.05 (1.04, 1.06) | 1.05 (1.04, 1.06) |

*Abbreviation:* *NC*, neck circumference; *BF%*, body fat percentage; *WHR*, waist hip ratio; *VAI*, visceral adiposity index; *LAP*, lipid accumulation product; *RMR*, resting metabolic rate.

^a^ Adjustment for age, sex, marital status, education, smoking, alcohol drinking, fruit intake, vegetable intake, work intensity, leisure-time physical activity, NC, WHR, BF%, VAI, LAP, and RMR.
